# Permutation Entropy Analysis to Intracranial Hypertension from a Porcine Model

**DOI:** 10.3390/e25020267

**Published:** 2023-01-31

**Authors:** Fernando Pose, Nicolas Ciarrocchi, Carlos Videla, Francisco O. Redelico

**Affiliations:** 1 Instituto de Medicina Traslacional e Ingeniería Biomédica, CONICET, Hospital Italiano de Buenos Aires, Instituto Universitario del Hospital Italiano de Buenos Aires, Ciudad Autónoma de Buenos Aires C1199ABB, Argentina; 2 Servicio de Terapia Intensiva de Adultos, Hospital Italiano de Buenos Aires, Ciudad Autónoma de Buenos Aires C1199ABB, Argentina; 3 Departamento de Ciencia y Tecnología, Universidad Nacional de Quilmes, Bernal B1876BXD, Argentina

**Keywords:** permutation entropy, intracranial pressure, intracranial compliance

## Abstract

Intracranial pressure (ICP) monitoring is commonly used in the follow-up of patients in intensive care units, but only a small part of the information available in the ICP time series is exploited. One of the most important features to guide patient follow-up and treatment is intracranial compliance. We propose using permutation entropy (PE) as a method to extract non-obvious information from the ICP curve. We analyzed the results of a pig experiment with sliding windows of 3600 samples and 1000 displacement samples, and estimated their respective PEs, their associated probability distributions, and the number of missing patterns (NMP). We observed that the behavior of PE is inverse to that of ICP, in addition to the fact that NMP appears as a surrogate for intracranial compliance. In lesion-free periods, PE is usually greater than 0.3, and normalized NMP is less than 90% and p(s1)>p(s720). Any deviation from these values could be a possible warning of altered neurophysiology. In the terminal phases of the lesion, the normalized NMP is higher than 95%, and PE is not sensitive to changes in ICP and p(s720)>p(s1). The results show that it could be used for real-time patient monitoring or as input for a machine learning tool.

## 1. Introduction

Intracranial pressure (ICP) is the result of the interaction of different components present within the cranial vault as well as the flow of the cerebrospinal fluid and the venous and arterial system inside and outside of the cranium [1]. In cases of acute brain injury, such as traumatic brain injury, preserved cerebral autoregulation allows for relatively preserved brain perfusion. Under conditions of elevated ICP, brain perfusion is compromised. A vasodilatory cascade may follow augmenting cerebral blood flow into the cranial vault at the expense of increasing the overall volume of the intracranial compartment and thereby increase ICP further. This phenomenon is particularly notorious as brain compliance is exhausted. The utility of ICP monitoring in its current form, while recommended by the brain trauma foundation is not without criticism. Evidently in the best trip trial, care focused on maintaining ICP at 20 mmHg or less was not superior to care based on imaging and clinical examination. However, ICP monitoring has been associated with lower mortality and improved outcomes in an international prospective observational cohort study [2,3]. These seemingly inconsistent observations may argue that the utility of ICP monitoring may lie in the information available but not exploited by simply aiming at ICP thresholds [4,5]. The ICP waveform has a lot of information that can be explored. In acknowledging the complexity of the intracranial dynamics, we approach the ICP waveform from a complexity science [6] perspective. One of the major contributions of this approach to the study of physiology is the possibility of thinking of a certain biosignal as the manifestation of an underlying complex system that in its turn behaves as a data-generating process [7,8]. It then follows that, by studying some characteristics of the biosignal, we can infer properties of the data-generating process and, therefore, better understand the underlying physiology. The other milestone in the use of the science of complexity in physiology is the so-called loss of complexity hypothesis (LCH) [9], which establishes that in the presence of aging, disease, or injury, the complexity of a physiological system, measured by its representative biosignal or bioimage, decreases, showing a decline in the ability of the system to adapt to injury [8]. This hypothesis has been verified in intracranial hemorrhage (ICH) patients [10,11,12,13,14,15,16,17,18,19,20]. Permutation entropy (PE) is a measure of complexity proposed by Bandt and Pompe for chaotic time series, in particular, in the presence of dynamic and observational noise [21]. Its estimation has some features that make it ideal for real-time patient monitoring, it is fast [22], which is very convenient for real-time implementation on an intensive care monitor; robust [23,24], it is very important to ensure the reliability of a real-time implementation on an intensive care monitor; it is distributional free [25], then no assumption about the physiological underlying generating process is needed, except for a very mild stationarity and it is bounded between the (0,1) interval. It is based on Shannon entropy [26,27] but analyses the ordering of neighboring values in a time series instead of using histograms to estimate the required probability distribution. As such, it is capable of detecting autocorrelation [23]. The PE was used several times in medicine, especially in the fields of epilepsy [25], anesthesia [28,29,30,31,32,33], cognitive neuroscience [34,35] and in the analysis of ICP waveforms [18,19]. In one study [18], the ICP waveform was analyzed from 6 episodes of intracranial hypertension extracted from the multiparametric database MIMIC III [36,37,38]. The PE estimation from the ICP waveform reproduced the LCH in the presence of ICH. In another paper [19], 69 signals from 33 patients with normal pressure hydrocephalus and 36 patients with secondary forms of normal pressure hydrocephalus were analyzed with similar overall results. In this work, we systematically analyze all PE characteristics of the ICP waveform from 4 animal experiments, with a total of 22 intracranial hypertension episodes each of which was reversible and one intracranial hypertension episode that was sustained to culminate in cessation of effective brain perfusion. It was performed in *Hospital Italiano de Buenos Aires*, in compliance with all ethical and legal regulations.

## 2. Brief Bibliographical Review

In order to put our contribution in context with previously published work, we conducted a literature search in Scopus and PubMed databases. The search in the Scopus database was done (in December 2022) using the following wide query TITLE-ABS-KEY (“Intracranial pressure”) AND TITLE-ABS-KEY (“entropy”) and the PubMed search using (“intracranial pressure”[Title/Abstract]) AND (entropy[Title/Abstract]). The search showed that the most frequently used entropies used to characterize the complexity of the ICP waveforms are approximate entropy [39], wavelet entropy [40], sample entropy [41], and permutation entropy [21].

This bibliographic search resulted in a total of 24 non-overlapping documents between the two databases analyzed. These results can be divided into 18 scientific articles in journals and 6 articles from conferences. The journal with the most publications is the *Journal of Neurotrauma* with four publications. Eight different databases have been used in these articles, of which, only one is publicly available. Only one database is comparable to ours, since it is a prospective experimental study performed in animals, the others are retrospective studies in humans and of these databases only 1 is publicly available. We summarized the major findings within the reviewed articles.

Concerning the animals database:

- In [10], a dataset of nine Sprague Dawley rats was exposed to cycles of hypotension (45–50 mmHg) for 15 min, followed by resuscitation and equilibration. Approximate entropy was calculated with parameters m=2 and r = 0.2 SD; its value resulted in the hypotension periods of 1.086 (±0.074), and after resuscitation, 1.242 (±0.087), which were statistically significantly different from those of the basal periods 0.691 (±0.212). However, there is no clear definition of the power of the statistical test used or why it uses these parameter values.

As for the human databases:

- In [42], data collected from 93 patients from 1998 to 2003 who were admitted to the Pediatric Intensive Care Unit of the Doernbecher Children’s Hospital (Oregon Health and Science University) were used. The data contribute to the casuistry of the decrease in complexity, measured using ApEnt, during periods of intracranial hypertension. The value of the parameters used in the estimation of ApEnt is not explicit.
- A retrospective analysis of 11 clinical intracranial hypertension episodes—a case series over a 30-month period from April 2000 to January 2003 is presented in [13]. They found that ApEn is lower during the intracranial hypertension period than during the stable and recovering periods.
- In [13], 11 episodes of intracranial hypertension from 7 subjects requiring ventriculostomy catheters for intracranial pressure monitoring and/or cerebral spinal fluid drainage were analyzed, with parameters
- In [11], 12 patients submitted to a preoperative study for the diagnosis of normotensive hydrocephalus. These patients showed oscillatory episodes of ICP in their nocturnal recordings. The ApEnt calculated on the ICP oscillatory state had a value of 0.23 (±0.06) was statistically different from the baseline period 0.41 (±0.10). In this case, there was also no consideration of the statistical power of the test and no clarification of the value of the parameters used to calculate ApEnt.
- In [13], a retrospective study of 11 ICP segments belonging to 7 traumatic brain-injured (TBI) pediatric patients is presented. ApEnt was calculated in three different periods: stable: 0.442 (±0.13), critical 0.26 (±0.07), and recovery 0.40 (±0.14) using the parameter values (m=1, r = 0.2 SD).
- The ICP time series was from four patients with hypertensive episodes using wavelet entropy. The results found are consistent with the general casuistry; the change in complexity is negative in the periods of the hypertension plateau. The wavelet entropy gives information that the wavelet energy is distributed differently in the basal condition than in the plateau, in which it is concentrated in low-frequency bands. This allows us to infer that the episodes of intracranial hypertension could be caused by a rearrangement of the oscillatory energy in the brain.
- A total of 120 ICP signals recorded during the infusion test were analyzed by wavelet entropy to characterize the different stages of the infusion test as a function of this entropy. The lowest value of the WE was found in the basal phase, a growth of this entropy was in the infusion phase, and the highest value was in the plateau phase, to finally decrease slightly in the recovery phase.
- In [16], a database of 325 patients with traumatic brain injury who were admitted to the Neurosciences Critical Care Unit, Addenbrooke’s Hospital, Cambridge, United Kingdom, between 2002 and 2010 was used. They reported that reduced complexity of ICP calculated in 3 h moving windows, might predict death in traumatic brain injured patients. The parameters used to calculate a multiscale of the sample entropy were not explicitly explained in [16], but they said that m=2 was used in [16].
- In [17], 30 patients were admitted to the neurocritical care unit at Addenbrooke’s Hospital between February 2005 and June 2006. The parameters used were m=1, r = 0.2 SD for SampEnt estimation. It also contributes to the casuistry of the decrease in complexity during periods of intracranial hypertension.
- In [19], 69 signals were selected from 33 patients with normal pressure hydrocephalus and 36 patients with a secondary form of normal pressure hydrocephalus. The possibility of discriminating patients with different types of hydrocephalus by infusion tests using permutation entropy was discussed. PE-ICP was calculated using m=5, τ=1. It was found that permutation entropy is able to distinguish between periods of “norm” and hydrocephalus, although it is not able to classify patients with different types of hydrocephalus.
- In [18], an analysis of the MIMIC-III waveform database matched subset containing all the complete data (vital signs, signals, clinical analysis, and annotations) of a subset of patients from the MIMIC-III database [36] is presented. The authors show that there is a PE-ICPd decrease in the period of hypertension; using the missing values technique, they showed that the increase in the period of hypertension is related to a decrease in the degree of freedom of the system to adapt, which would be an indication that the complexity decreased by the failure of the mechanisms of cerebral autoregulation, according to the theory proposed in [8].

There are also other papers that analyzed the ICP time series as a function of some entropic quantifier; but they either used simulated data or the sources of the data were not clearly identified, as in [43,44]. This is the context of our analysis, which presents a proof of concept of the plausibility of using permutation entropy and the number of missing patterns for monitoring the neurocritical patient. Most datasets are of similar sizes to ours. Given their retrospective nature in humans, all the datasets were not obtained under controlled conditions, so there are many variables (e.g., comorbidities, drug use, therapeutic strategies, etc.) that may contribute to the modification of ICP complexity. Our data come from an experiment under controlled conditions, in the same way as [10], so we can assure that the changes in complexity obtained and concomitantly in the number of missing patterns are only due to the modifications of the system caused by the elevation of ICP. It is important to note that, unlike the previously analyzed datasets, we have two extreme stages, the first stage where there is no lesion in the brain so we could consider that the ICP has natural complexity, and the determination of the ICP baseline complexity could be useful for other neurodegenerative pathologies, such as Alzheimer’s, as it was hypothesized that high intracranial pressure could play a role in the pathogenesis of Alzheimer’s [45,46,47], and the most extreme stage, the third stage, where there is a condition of cerebral circulation arrest. All other experiments seem to have been developed in conditions similar to our second stage, although not in a controlled experiment, except for [10].

## 3. Materials and Methods

### 3.1. Experimental Data

The animal model used in this paper was approved under number 0007/19 by the Institutional Committee for the Care and Use of Laboratory Animals belonging *Instituto de Medicina Translacional e Ingeniería Biomedica (IMTIB, HIBA-IUHIBA-CONICET)* and it met all of the regulations required in the Argentine legislation. Summarizing, 5 previously healthy 6-month-old landrace pigs with an average weight of 25–30 kg under general anesthesia, placed in ventral decubitus, were selected to participate in the experiment, which consisted of three stages:

- *Anesthesia stage*: General anesthesia was induced through an atrial vein with 6 mg/kg fentanyl, 4 mg/kg propofol, and 1.2–1.5 mg/kg rocuronium. Intubation was performed with a 7.0 mm cuffed tube. Anesthesia was maintained with a continuous intravenous infusion of propofol 0.25–0.30 mcg/kg/min, remifentanil 0.5 mcg/kg/min, and pancuronium 0.04 mgmg/kg/h. Mechanical ventilation was performed in a pressure-controlled mode with a positive pressure at the end of the expiration of 5 cm H_2_O and a FiO_2_ of 0.40. The respiratory rate and tidal volume were adjusted to maintain normocarbia throughout the experiment, which was controlled with a spirometer. Normovolemia was maintained by infusing a complete electrolyte solution at a rate of 10–15 mL/kg/h.
- *First stage (baseline period)*: In this stage, after proper anesthesia, the ICP catheter was placed through a burr hole 20 mm anterior to the coronal suture, and 15 mm from the sagittal suture (midline) and, once the correct measurement of ICP was obtained, a duration of 5 min was recorded.
- *Second stage (reversible intracranial hypertension)*: An 8 French Foley catheter (FC) was placed through another burr hole 20 mm anterior to the coronal suture and a 15 mm sagittal suture contralateral to the ICP catheter. After this, several reversible intracranial hypertension episodes were generated using 0.9% saline in the FC with a continuous infusion pump to control the volume and rate of infusion. After reaching the target ICP value—after 5 min—deflation was carried out progressively at a rate of 1 mL/min.
- *Third stage (cerebral circulatory arrest)*: Intracranial hypertension was induced using the same Foley catheter and the infusion pump to a target cerebral perfusion pressure of less than 10 mmHg for no less than one hour and an EEG compatible with electrocerebral silence.

At the end of the experiment, the animals were sacrificed via an IV overdose of propofol (20 mg/kg) and fentanyl (10 mg/kg) followed by 40 mL 19.1% potassium chloride solution. A complete and detailed narrative of the experiment was previously published in [48].

The ICP time series were downloaded from a Phillips Intellivue MP40 multiparametric monitor (Philips Healthcare, Inc., Andover, MA) during the experiment (first, second, and third stages) using custom-made software based on the open-source application VSCapture [49]. VSCaptureMP uses the C# .NET/Mono programming platform, and the data capture is based on an event-triggering programming paradigm at the corresponding port. It currently uses either UDP/IP protocol via the LAN or the MIB/RS232 port on the monitor for data logging. It is freely available from https://sourceforge.net/projects/vscapture/, accessed on 3 October 2022), It has been used several times in previously published research [48,50,51,52,53,54,55,56]. The sampling frequency for ICP was 200 Hz. After downloading, the ICP signal was filtered using a band-stop Butterworth filter to remove the power-line noise frequency at 50 Hz. Finally, a low-pass Butterworth filter with a cut-off frequency 60 Hz was applied in order to remove high-frequency noise. The filter order was 4 for all filters used. All data are downloaded to a csv (comma-separated value) format and are accessible from the corresponding author given a reasonable request. All computations were done using Python libraries. Unfortunately, during the first experiment, there were problems in the interface between the monitor and the software, so the first pig experiment was excluded from this paper because its sampling frequency (1 Hz) is not suitable for the tools proposed here.

**Figure 1:**
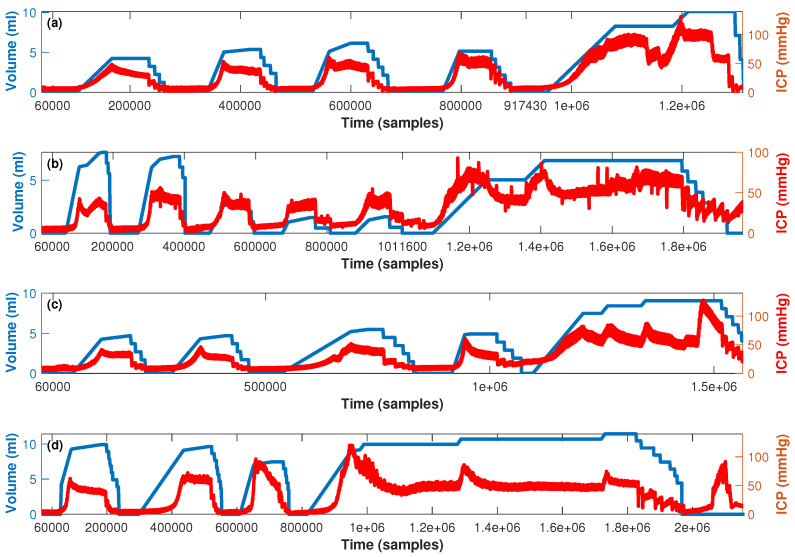
Timeline for the experiment presented in [48]. For each experimented pig (**a**–**d** for pigs one to four respectivelly): the balloon volume is represented with the blue line and the ICP time series with the red line.

**Figure 2:**
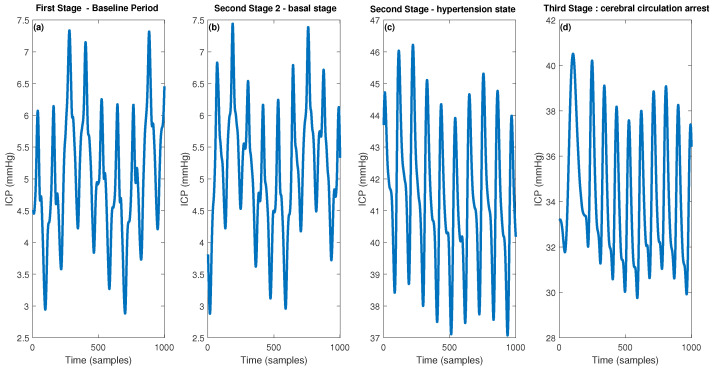
Representatives of 1000 samples (5 s) of the ICP time series in the three stages of the experiment, (**a**) First Stage—Baseline Period, (**b**) Second Stage 2—basal stage, (**c**) Second Stage—hypertension state and (**d**) Third Stage—cerebral circulation arrest.

In Figure 1, the timeline for the four pigs analyzed is presented. Emptying and filling of the balloon generated by the French Foley catheter is presented along the blue line (left y-axis) and the ICP time series is presented along the red line (right y-axis). In Figure 2, 4 examples of 5 s (1000 samples) from the 3 stages are presented. Figure 2a shows the ICP during the first stage when no manipulation with the Foley catheter was performed yet. This can be considered the baseline state of the experiment. Figure 2b shows the ICP during the second stage of the experiment when the balloon volume was equal to 0 and ICP was normal (around 5 mmHg). Figure 2b shows the ICP during the second stage of the experiment when the balloon volume is different from 0 and ICP is extremely high (around 42 mmHg). Figure 2c shows the ICP during the third stage of the experiment where cerebral circulation arrest was achieved, and ICP was also very high (around 35 mmHg). The current medical recommendation [57] is to perform some treatment when the ICP exceeds 20 mmHg, i.e., from this value, ICP elevation can be considered as high and dangerous.

### 3.2. Intracranial Compliance

The relationship between intracranial pressure and intracranial volume, which we call intracranial compliance (ICC), is based on the Monro–Kellie doctrine [58,59], which states that the intracranial contents are composed of three cavities—the cerebrospinal fluid (CSF), the brain, and the blood volume—so the ability of the intracranial system to adapt to new changes in volume in any of these compartments is the ICC. This is a dynamic property of the brain that is simply defined as the derivative of the intracranial volume with respect to the intracranial pressure, as follows

(1) $$\mathrm{ICC}=\frac{dVolume}{dICP}$$

It is currently hypothesized [60] that in addition to the Monro–Kellie theory, there is a nonlinear interaction of many factors, including CO_2_ partial pressure, endothelial function, brain hydration, and metabolism. The possibility of determining intracranial compliance in actual patients in a noninvasive way involves active research [61].

### 3.3. Permutation Entropy

Within this section, we briefly describe the permutation entropy calculation. The entropy of a system can be measured using the Shannon formulation [26,27], $S\left(f\right)=\int_{\Delta }flog\left(f\right)dx$, where the integration envelopes all possible states *f*. In the case of a discrete-state system, such as time series data, the discrete Shannon entropy would be $S\left(P\right)=\Sigma_{i=1}^{N}p_{i}log\left(p_{i}\right)$ and a normalized version is

(2) $$H\left[P\right]=\frac{S(P)}{S_{max}}$$

where the denominator $S_{m}ax=S\left[P_{e}\right]=lnN$ is the maximum entropy obtained by a uniform probability distribution $P_{e}=p_{i}=\frac{1}{N},i=1,\cdots ,N$, and $P=p_{1},\cdots ,p_{N}$ is the vector containing the probabilities that the systems are in the i-th state.

There is no universal way to compute *P* in Ec. 1. Spectral entropy, wavelet entropy, or permutation entropy, were used, just to mention a few. One of the methods proposed to compute **P** is the symbolization proposed by Bandt and Pompe in [21] (BP-symbolization). It consists of a translation from the times series onto a symbolic sequence, as explained below.

Let $S_{\left(m⩾3\right)}$ the set form by all possible permutations of order *m*, $s_{i}=\left(i_{1},i_{2},\ldots ,i_{m}\right)\in S_{m}$, i.e, $S_{m}$ is the symbol set. Each $s_{i}\in S_{m}i$ is called a symbol, a pattern, or a motive, and the cardinality of $S_{m}$ is m!, as each $s_{i}$ is unique. For example, if we set m=3, the cardinality of $S_{3}$ is 3!=6, i.e., the set $S_{3}$ contains six symbols and becomes $S_{3}=\left(123),(132),(231),(213),(312),(321\right)$. Once the symbol set is stated, one should map the time series onto a sequence of symbols from this set. Consider for the time series $X_{T}=x_{1},\ldots ,x_{T}$ and its m-dimension embedding representation as $X_{t}^{\left(m,\tau \right)}=(x_{t},x_{\left(t+\tau \right)},\ldots ,$$x_{\left(t+(m-1)\tau \right)})$, without loss of generality, we take τ=1. Then the vector $X_{t}^{m}$ can be mapped to a symbol vector $s\in S_{m}$. This mapping should be defined in a way that preserves the desired relation between the elements $x_{t}\in X_{t}^{m}$ and all t∈T that share this pattern have to be mapped to the same s.

We will exemplify all the procedures. Let $X_{7}=3,6,8,9,5,10,2$ and the embedding dimension m=3, so the T−(m−1)=7−(3−1)=5 embedded vectors $X_{t}^{m=3}$ are $X_{1}^{m=3}=\left(3,6,8\right),X_{2}^{m=3}=\left(6,8,9\right),X_{3}^{m=3}=\left(8,9,5\right),X_{4}^{m=3}=\left(9,5,10\right)$, and $X_{5}^{m=3}=\left(5,10,2\right)$ and the mapping was

(3) $$\begin{array}{ccc} X_{1}^{\left(3\right)} & = & \left(\overset{1}{3},\overset{2}{6},\overset{3}{8}\right)\underset{rearrange}{\to }\left(\overset{3}{1},\overset{6}{2},\overset{8}{3}\right)\underset{\mathrm{map}\;\mathrm{onto}}{\to }s_{1}=123\\ X_{2}^{\left(3\right)} & = & \left(\overset{1}{6},\overset{2}{8},\overset{3}{9}\right)\underset{rearrange}{\to }\left(\overset{6}{1},\overset{8}{2},\overset{9}{3}\right)\underset{\mathrm{map}\;\mathrm{onto}}{\to }s_{2}=123\\ X_{3}^{\left(3\right)} & = & \left(\overset{1}{8},\overset{2}{9},\overset{3}{5}\right)\underset{rearrange}{\to }\left(\overset{5}{3},\overset{8}{1},\overset{9}{2}\right)\underset{\mathrm{map}\;\mathrm{onto}}{\to }s_{3}=312\\ X_{4}^{\left(3\right)} & = & \left(\overset{1}{9},\overset{2}{5},\overset{3}{10}\right)\underset{rearrange}{\to }\left(\overset{5}{2},\overset{9}{1},\overset{10}{3}\right)\underset{\mathrm{map}\;\mathrm{onto}}{\to }s_{4}=213\\ X_{5}^{\left(3\right)} & = & \left(\overset{1}{5},\overset{2}{10},\overset{3}{2}\right)\underset{rearrange}{\to }\left(\overset{2}{3},\overset{5}{1},\overset{10}{2}\right)\underset{\mathrm{map}\;\mathrm{onto}}{\to }s_{5}=312 \end{array}$$

Hence, the symbol sequence generated by $X_{7}$ is $s_{1},s_{1},s_{5},s_{4},s_{5}$ Now it is time to count the probability of each $s_{i}$ in order to obtain the required $p_{i}$ in Ec. 1. From the example above, $p_{1}=p\left(s_{1}\right)=\frac{2}{5},p_{2}=p\left(s_{2}\right)=0,p_{3}=p\left(s_{3}\right)=0,p_{4}=p\left(s_{4}\right)=1/4,p_{5}=p\left(s_{5}\right)=\frac{2}{5}$. For a more in-depth explanation of the possible ways of calculating the patterns, the interested reader can refer to [62].

Finally, it is important to note that in our example, there are two symbols that do not appear, $s_{2}$ and $s_{3}$. That means, their probability is equal to 0. In many actual and simulated time series, this phenomenon is present. The number of missing (or forbidden) patterns (NMP), i.e., the number of symbols with 0 probability of occurrence in the time series spam, has been used several times, for example, as a chaos signature [63] or with a physiological meaning in [18]. In our example, the NMP is equal to 2 because patterns $s_{2}$ and $s_{3}$ do not appear in the time series and have both 0 probabilities. We have to note that it is the $p_{i}$ different from 0 that drives the PE value and not the NMP value, the last one is not directly related to the PE value estimated, to our knowledge. The NMP is a by-product of BP-symbolization. In this paper, we try to interpret the NMP value in terms of intracranial physiology.

Keeping in mind that the seminal paper on PE [21] recommends exploring the values of {m=3,⋯,7} we first evaluate PE with each of these embedding dimensions, with a window size of 5m! (and τ=1, to avoid possible confusion with the time scale). We find visually that the value of m=6 is a suitable compromise between adequate tracking of the ICP signal and computational cost since when we use m=7 we need to estimate the probabilities of 5040=7! different symbols, in contrast when, m=6 which only needs 720=6! [64]. The computational cost is important when one has in mind the practical use of permutation entropy, especially if this use could be intended for hospitals with scarce computational resources as in developing countries [65].

## 4. Results

**Figure 3:**
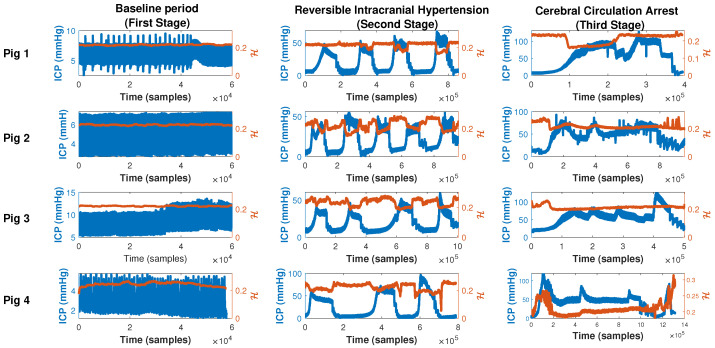
Dynamics of the ICP (blue line, left y-axis) superposed with its respective permutation entropy (red line, right y-axis) computed on a sliced window of length 3600 samples with a 1000 samples shift and using m=6,τ=1 as the values of parameters for all stages within the experiment (columns) and each pig (rows).

Figure 3 shows the ICP waveform (blue line, left y-axis, mmHg) for the four pigs (one pig in each row) superimposed on the PE (red line) computed over each window for all stages in columns. It is shown that during the baseline period (first column), PE remains almost constant near 0.2 for all pigs. In addition to this, in most episodes within stage two (second column), across all pigs, PE has an inverse evolution to ICP. As ICP increases, PE decreases suggesting a more predictable (less complex) state. As ICP returns to baseline values, PE increases suggesting increasing complexity, or more adaptability [8,18]. Interestingly, this is not the case during the third stage. During the third stage (third column), PE does not follow ICP fluctuations. This is best appreciated in pigs 3 (third row) and 4 (fourth row). During these periods of refractory intracranial hypertension (stage 3), ICP fluctuations are not associated with PE changes. PE remains almost constant or has an inverse behavior to the stage described earlier, this is true except for the first part of stage 3 (column 3) in pig 2 (row 2), where the cerebral circulation arrest is achieved only at the end of this stage in this pig.

**Figure 4:**
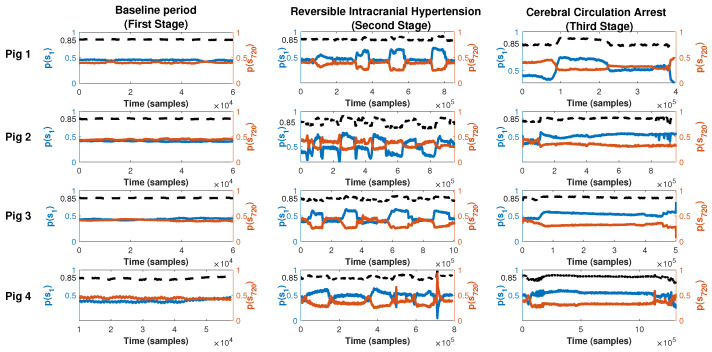
Time evolution of $p(s_{1})$ (blue line, left axis) and $p(s_{720})$ (red line, right axis) for all stages within the experiment (columns) and each pig (rows). The sum of the two probabilities is shown as a dotted black line and is read on the left axis.

We also analyzed the composition of the vector p which contributes to PE, Ec. 1. Figure 4 shows the probability for $s_{1}$ ($p(s_{1})$ blue line, left y-axis) and for $s_{720}$ ($p(s_{720})$, red line, right y-axis) for all pigs (rows) and the three stages (columns). Both vectors contribute 0.85% to PE but in intracranial hypertension periods, the contributions of these two patterns increase to 90%, even in a reversed manner, as $p(s_{720})$ becomes greater than $p(s_{1})$, reversing this relation in every transition between baseline ICP values and intracranial hypertension.

**Figure 5:**
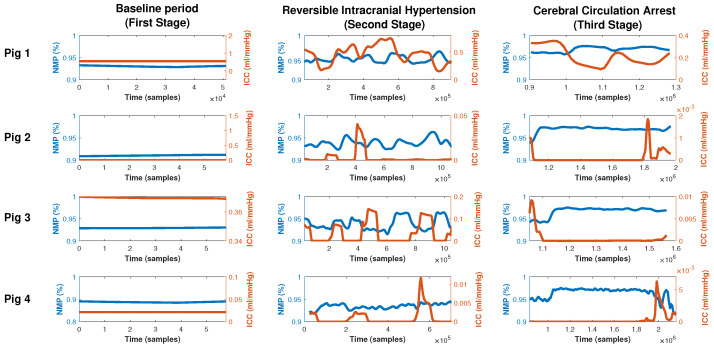
Intracranial Compliance (red line, right axis) superposed with its respective number of missing patterns (%) (blue line, left axis) computed on a sliced window of length 3600 samples with a 1000-sample shift and using m=6,τ=1 for the Bandt and Pompe symbolization for all stages within the experiment (columns) and each pig (rows). We have normalized the NMP to obtain a scale between 0 and 1 and not to 0 and 720 as the former number are easy to visualize.

The NMP is the number of patterns that do not appear in an actual time series and may be related to the ability of a physiological system to adapt to environmental change [18]. We computed normalized NMP for each window and normalized them over 720 in order to obtain a scale bounded between 0 and 1. Figure 5 shows the normalized NMP (red line) along with the ICP waveform (blue line, left y-axis) and ICC (red line, right y-axis) for the four pigs analyzed. In stage 1 (first column), NMP remains almost constant with similar values between pigs (0.92, 0.91, 0.92, and 0.88 for pigs 1, 2, 3, and 4 respectively). During the second stage (second column), ICC and NMP have opposite behavior, one ICC growth, NMP decreases, and vice versa. is correlated with ICP; when ICP increases, the proportion of NMP also increases. However, this association is not as robust as the relationship with PE suggesting that the later fluctuation is not being fully explained solely by an increasing NMP. This is expected since the MPN could show the adaptive capacity of the system and the ICC is the ability of the cranial-spine system to adapt to volume additions. In pig 2 (first row), ICC behaves differently from the rest of the pigs because the ICC does not reach values as low as in the others. This is because the brain was more compliant and was better able to contain volume changes.

**Figure 6:**
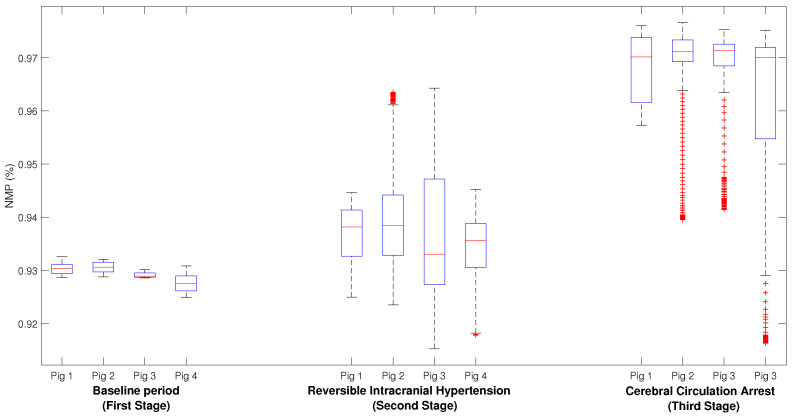
Box plot comparing the NMP (%) during each stage within the experiment for all pigs.

Figure 6 shows a comparison of the NMPs for all stages between all pigs. All pigs have similar medians within each stage. For stage one, the median NMPs % are 0.93, 0.93 0.92, and 0.92 for pigs 1, 2, 3, and 4, respectively, for stage two, the median NMPs % are 0.94, 0.94, 0.93, and 0.94 for pigs 1, 2, 3, and 4, respectively, and finally, for stage 3, the NMP was around 0.97 for all pigs.

## 5. Discussion

We can define a complex biological system as one that is sensitive to changes in initial conditions and reacts in an adaptive way to changes in the environment. When this capacity is lost, we can postulate that the complexity of the system is also affected. It has been hypothesized that complexity decreases in the presence of a stressor [8]. To the best of our knowledge, this is the first time that permutation entropy has been applied in an intracranial hypertension experiment. In this model, we see that periods of intracranial hypertension have lower PEs and, therefore, lower complexity states, affirming the loss of complexity hypothesis. During a cerebral circulatory arrest, PE remains constantly behaving differently from what is observed during the reversible period of intracranial hypertension (stage 2). In stage 3, the brain is irreversibly injured and compliance depleted manifesting as irreversible intracranial hypertension. ICP is a reflection of these mechanisms and the fact that PE behaves differently in these two stages supports this understanding.

Furthermore, during intracranial hypertension periods, PE decreases. A decrease in PE could be associated with an increase in the number of missing patterns speaking to lower degrees of freedom in the system and lower adaptability of the cerebrovascular system. Normalized NMP in the “healthy” pig is within the (0.85, 0.88) range. In contrast, during the hypertension episodes, the range is (0.90,0.98). If NMP indeed reflects the ability of a system to adapt to the environment, it is (0.85, 0.88) the lowest value to quantify the normal brain’s ability to change. The possibility of using the normalized NMP as a surrogate for brain compliance is very important. Since brain compliance is an indicator of the ability of the brain to withstand new insults (increased volume) and also cannot be measured noninvasive and in real-time in hospitalized patients, having the possibility of estimating it by means of an indicator could have a great impact on monitoring patients. Regarding stage three, it is interesting to note that the mean normalized NMP is almost constant at a high value of 0.96, no matter the ICP fluctuation, and it is similar across all pigs. This feature may be characteristic of ICP when severe and terminal brain hypo-perfusion heralds imminent cerebral circulatory arrest.

To understand the PE fluctuations, it is important to mention that the vector that contributes to the entropy (**P**) has two major components: $s_{1}$ and $s_{720}$. In the baseline periods, $p(s_{1})$ is greater than $p(s_{720})$ and both contribute 80% of PE but in intracranial hypertension periods, the contribution of these two patterns rises to 90% and is reversed: $p(s_{720})$ becomes greater than $p(s_{1})$. This reversion could be seen as a precursor of intracranial hypertension. It is a similar phenomenon as in wavelet entropy, where accommodation of wavelet relative energy is found in the intracranial hypertension periods, but with one difference, which is the energy, reorganization occurs in the lowest frequency patterns. The fact that $s_{720}$ has more energy, and more probability, than $s_{1}$ in the intracranial hypertension periods, may reflect the fact that the system tries to recover the baseline homeostatic ICP state. In the second part of the experiment, this feature, i.e., $p\left(s_{720}\right)>p\left(s_{1}\right)$, is also observed no matter the value and the behavior of ICP, suggesting a new equilibrium state. From the point of view of the clinical implication, Lu et al. [15] showed that the loss of the complexity of the intracranial pressure signal correlates with worse outcomes. So, we postulate that in monitoring intracranial pressure, we need to move beyond the mere absolute value of ICP to changes in the complexity of the signal as the latter provides important information about the status of cerebral compliance and may be determined in real time.

## 6. Conclusions

ICP waveform analysis yields significant and valuable information that sheds light on intracranial physiology and dynamics. Our dataset contains an injury-free stage, the EP found at that stage can be considered an estimate of the natural complexity of the brain. This is very important, not only for monitoring in intensive therapy but also for other neurodegenerative diseases, such as Alzheimer’s disease [47]. We have related MPN to intracranial compliance, so that the former could be a surrogate of the latter. Monitoring intracranial compliance is very important in critical neurological therapy. Finally, we have shown that EP can characterize ICP complexity in episodes of intracranial hypertension (Stage 2) and cerebral circulatory arrest (Stage 3).

We present a set of features extracted from the ICP waveform that can be used along with the mean ICP value to monitor the injured brain. A combination of the set (PE, NMP, and ($p(s_{1})$,$p(s_{720})$)) could give a better understanding of brain compliance than the ICP mean value itself. Although these characteristics are different in each pig analyzed, we can make some generalizations. In periods free of injury, PE is typically higher than 0.3, the normalized NMP is lower than 90%, and $p\left(s_{1}\right)>p\left(s_{720}\right)$. Any departures from these values could be a possible warning of disrupted physiology. Similarly, in terminal stages of injury (stage 3 in this experimental model) the normalized NMP equal to or greater than 95%, PE is not sensitive to ICP changes and in most cases, $p\left(s_{720}\right)>p\left(s_{1}\right)$. Between these two extreme conditions is stage two, where the most important feature is the variation of PE in association with ICP fluctuation and the fact that NMP ranges between 90 and 95%. We have to note at this point that brain compliance cannot be monitored noninvasively, which makes the hypothesis that it could be monitored by means of NMP a great contribution to clinical practice. Furthermore, the characterization of ICP time series complexity by permutation entropy could serve in the future as a guide for the monitoring and treatment of intracranial hypertension. This observational and descriptive study should be seen as a proof of concept that initiates the way for the use of permutation entropy and the number of missing patterns in the Bandt and Pompe symbolization in the monitoring of the neurocritical patient. Further clinical research and validation are necessary to set the cut-off points for PE and the normalized NMP, before the application of PE to ICP monitoring at the bedside.

## Data Availability

The data that support the findings of this study are available from the corresponding author upon reasonable request.
